# The phosphorylation of endogenous Nedd4-2 In Na^+^—absorbing human airway epithelial cells

**DOI:** 10.1016/j.ejphar.2014.03.005

**Published:** 2014-06-05

**Authors:** Noor A.S. Ismail, Deborah L. Baines, Stuart M. Wilson

**Affiliations:** a Division of Cardiovascular and Diabetes Medicine, Medical Research Institute, College of Medicine, Dentistry & Nursing, University of Dundee, Dundee DD1 9SY, UK; b Biochemistry Department, Faculty of Medicine, University Kebangsaan Malaysia, Jalan Raja Muda Abdul Aziz, 50300 Kuala Lumpur, Malaysia; c Biomedical Sciences Research Centre, St Georges University of London, Cranmer Terrace, London, UK; d Wolfson Research Institute, School of Medicine, Pharmacy and Health, Durham University Queen׳s Campus, Stockton on Tees, TS17 6BH, UK

**Keywords:** cAMP, cyclic 3′5′ adenosine monophosphate, CREB, cyclic AMP response element binding protein, EDTA, ethylene diamine tetra acetic acid, ENaC, epithelial sodium channel, *I*_Amil_, amiloride-sensitive component of the transepithelial current, IBMX, isomethyl butyl xanthine, Nedd4-2, neural precursor cell expressed, developmentally down-regulated protein 4-2, NDRG1, protein encoded by n-myc downstream regulated gene 1, PAGE, polyacylamide gel electrophoresis, PKA, cyclic adenine nucleotide-dependent protein kinase, SDS, sodium dodecyl sulphate, SGK1, serum and glucocortcoid-inducible kinase 1, s.e.m., standard error of the mean, Epithelial Na^+^ channel, Serum and glucocorticoid regulated kinase 1, Protein kinase A, Pulmonary Na^+^ absorption, Cellular signalling

## Abstract

Neural precursor cell expressed, developmentally down-regulated protein 4-2 (Nedd4-2) mediates the internalisation / degradation of epithelial Na^+^ channel subunits (α-, β- and γ-ENaC). Serum / glucocorticoid inducible kinase 1 (SGK1) and protein kinase A (PKA) both appear to inhibit this process by phosphorylating Nedd4-2-Ser^221^, -Ser^327^ and -Thr^246^. This Nedd4-2 inactivation process is thought to be central to the hormonal control of Na^+^ absorption. The present study of H441 human airway epithelial cells therefore explores the effects of SGK1 and / or PKA upon the phosphorylation / abundance of endogenous Nedd4-2; the surface expression of ENaC subunits, and electrogenic Na^+^ transport. Effects on Nedd4-2 phosphorylation/abundance and the surface expression of ENaC were monitored by western analysis, whilst Na^+^ absorption was quantified electrometrically. Acutely (20 min) activating PKA in glucocorticoid-deprived (24 h) cells increased the abundance of Ser^221^-phosphorylated, Ser^327^-phosphorylated and total Nedd4-2 without altering the abundance of Thr^246^-phosphorylated Nedd4-2. Activating PKA under these conditions did not cause a co-ordinated increase in the surface abundance of α-, β- and γ-ENaC and had only a very small effect upon electrogenic Na^+^ absorption. Activating PKA (20 min) in glucocorticoid-treated (0.2 µM dexamethasone, 24 h) cells, on the other hand, increased the abundance of Ser^221^-, Ser^327^- and Thr^246^-phosphorylated and total Nedd4-2; increased the surface abundance of α-, β- and γ-ENaC and evoked a clear stimulation of Na^+^ transport. Chronic glucocorticoid stimulation therefore appears to allow cAMP-dependent control of Na^+^ absorption by facilitating the effects of PKA upon the Nedd4-2 and ENaC subunits.

## 1 Introduction

The controlled absorption of Na^+^ from the liquid film covering the lung / airway epithelia is vital to the integrated functioning of the respiratory tract and occurs via a ‘leak-pump′ mechanism in which the overall rate of Na^+^ absorption is limited by the rate of apical Na^+^ entry though epithelial Na^+^ channels (ENaC). Glucocorticoids are clearly important to the induction and maintenance of this Na^+^ absorbing phenotype and synthetic glucocorticoids are therefore used in the clinical management of conditions, such as respiratory distress and pulmonary oedema, that involve dysfunction of pulmonary Na^+^ absorption (Barker and Olver, 2002; Boucher**,** 2004; 2007; Matthay et al., 2002; Olver et al., 2004; Wilson et al., 2007). However, despite their clear importance, the mechanisms that allow these hormones to control pulmonary Na^+^ transport are not understood fully (Barnes, 2011; Matthay et al., 2002; Olver et al., 2004).

Epithelial Na^+^ channels are composed of three subunits (α-, β- and γ-ENaC) that contain proline-rich domains which provide binding sites for neural precursor cell expressed, developmentally down-regulated protein 4-2 (Nedd4-2). The binding of Nedd4-2 to these sites targets the ENaC channel complex for internalisation and degradation and, in unstimulated cells, this internalisation/degradation process is thought to limit the surface abundance of ENaC and thus restrict the rate of Na^+^ transport (Debonneville et al., 2001; Snyder et al., 2004a; Snyder et al., 2002). Nedd4-2 gene deletion thus disrupts lung function by inducing excessive Na^+^ absorption that reflects an increase in the amount of α-, β- and γ-ENaC at the cell surface (Boase et al., 2011; Kimura et al., 2011). However, it is also clear that Nedd4-2 can be phosphorylated at Ser^221^, Ser^327^ and Thr^246^ by serum / glucocorticoid-inducible kinase 1 (SGK1) and by protein kinase A (PKA). Since this phosphorylation of Nedd4-2 blocks the interaction with ENaC, it is thought to cause an increase in the amount or α-, β- and γ-ENaC present at the cell surface. Moreover, since SGK1 and PKA are under hormonal control, the regulated phosphorylation / inactivation of Nedd4-2 is thought to allow control over the rate of Na^+^ absorption (Baines, 2013; Boase et al., 2011; Debonneville et al., 2001; Flores et al., 2003, 2005; Kimura et al., 2011; Morris and Schafer, 2002; Snyder et al., 2002, 2004a, 2004b; ). However, few authors have quantified the phosphorylation of endogenous Nedd4-2 (see Flores et al., 2005) and the evidence supporting this model is mainly derived from heterologous expression systems (Chandran et al., 2011; Debonneville et al., 2001; Snyder et al., 2002, 2004a). To clarify the mechanisms that allow hormones to regulate pulmonary Na^+^ transport, we now use newly-developed, phospho-peptide specific antibodies to study the effects of SGK1 and PKA upon the phosphorylation of endogenous Nedd4-2 in a Na^+^ absorbing human airway epithelial cells (H441) (Althaus et al., 2010; Clunes et al., 2004; Lazrak and Matalon, 2003; Thomas et al., 2004; Watt et al., 2012).

## 2 Materials and methods

### 2.1 Antibodies

The polyclonal antibodies against Nedd4-2 were all produced within the MRC Protein Phosphorylation Unit (MRC-PPU), College of Life Sciences, University of Dundee and were generously made available by Professor Sir Philip Cohen and Professor Carol MacKintosh. The antibody against Ser^221^- (antibody identification number: DSTT-S754A) phosphorylated Nedd4-2 was generated against a phospho-peptide (SRLRSCS⁎VTDTVA, where ⁎ indicates phosphorylated residue) corresponding to the residues 215–227 of human Nedd4-2. Similarly, the antibodies against the Ser^327^- (DSTT-S755A) and Thr^246^-phosphorylated (DSTT-S753A) Nedd4-2 were directed against residues 317–331 (CEPQIRPRSLS⁎SPTV) and 240–252 (GRARSST⁎VTGGEE) respectively. Initial studies undertaken by Dr James Hastie (Antibody production unit, MRC Protein Phosphorylation Unit, University of Dundee) confirmed that these phospho-peptide specific antibodies displayed minimal affinity for unphosphorylated, GST-Nedd4-2 but did bind to GST-Nedd4-2 that had been phosphorylated in vitro using recombinant protein kinase B or SGK1. The antibodies therefore recognise the Ser^221^-, Ser^327^ and Thr^246^-phosphorylated forms of human Nedd4-2. The antibody against total Nedd4-2 (DSTT-S741B) was raised in sheep against recombinant His-tagged Nedd4-2 expressed in insect cells. Antibodies against total (DSTT-S276B) and Thr^346/356/366^-phosphorylated (DSTT-S231B) forms of the protein encoded by N-myc-downstream regulated gene 1 (NDRG1) were also from the MRC-PPU (Murray et al., 2004) whilst the antibodies against the total and Ser^133^-phosphorylated cyclic AMP response element binding protein (CREB) were from Cell Signalling (Hertfordhire, UK). The antibody against *α*-ENaC was a gift from Prof. Rick Boucher (Cystic Fibrosis Center, University of North Carolina, USA), whilst the β-ENaC was from Santa Cruz Biotechnology (Heidelberg, Germany; product code SC-21013) and the γ-ENaC antibody from Sigma (Poole, Dorset UK; product code E4902).

### 2.2 Cell culture

Stocks of H441 human airway epithelial cells were maintained in RPMI medium supplemented with 8.5% foetal bovine serum, 8.5% newborn calf serum (Life Technologies Ltd, Paisley, UK), 2 mM glutamine, 5 µg ml^−1^ transferrin, 5 ng ml^−1^ selenium and an antibiotic / antimycotic mixture (Sigma Chemical Co., Poole, Dorset). For experiments, cells removed from culture flasks using trypsin/ethylene diamine tetra acetic acid (EDTA) were plated onto 6 well plates (analysis of extracted protein) or Costar “Snapwell” membranes (electrometric experiments) and maintained in this standard growth medium for 5–7 days. Twenty four hours before being used in experiments, this medium was replaced with a fully-defined medium identical to that described above except that (*i*) it contained 20 nM insulin and (*ii*) the serum components were replaced with FBS (8.5%) that had been dialysed (molecular weight cut<10 KDa) to remove hormones/growth factors (Life technologies, ltd., product code 26400044). “Glucocorticoid-deprived” cells were maintained in this nominally hormone-free medium for ~24 h before being used in experiments whilst “dexamethasone-treated” cells were maintained in medium supplemented with 0.2 µM dexamethasone for 3 h (brief exposure) or 24 h (chronic exposure). In some experiments cells were exposed to a cocktail of compounds (10 *µ*M forskolin, 100 µM iso-butylmethylxanthine and 1 mM N-6-2-O-dibutyryladenosine 3′5′-cyclic monophosphosphate, referred to as cAMP agonists) that activate cAMP-dependent signalling pathways.

### 2.3 Expression/phosphorylation of Nedd4-2

Control/stimulated cells were washed with ice-cold phosphate buffered saline and scraped into ice-cold lysis buffer containing protease and phosphatase inhibitors (1% Triton; 50 mM Tris–HCl, pH 7.5; 1 mM EGTA; 1 mM EDTA; 1 mM Na orthovanadate; 10 mM glycerol phosphate; 50 mM NaF; 5 mM Na pyrophosphate; 270 mM sucrose; 0.1% β-mercaptoethanol; 1 Roche Mini Protease Inhibitor tablet per 10 ml). The lysates were then ultrasonicated and their protein contents determined using Bradford reagent. Aliquots (1 mg) of extracted protein were then cleared by incubation (1 h, 4 °C) with uncoated Sepharose G beads and the cleared lysates then exposed to Sepharose G beads coated with 10 µg of anti Nedd4-2. After 1 h incubation at 4 °C with continual agitation, the beads were precipitated by centrifugation; washed sequentially with high (500 mM NaCl, 50 mM Tris / HCl; pH 7.5) and low (50 mM NaCl, 1 mM EDTA, 0.1% β-mercaptoethanol; Tris / HCl, pH 7.5) salt buffers, and adherent proteins recovered by heating the beads to 95 °C in the sample buffer used for sodium dodecyl sulphate-polyacylamide gel electrophoresis (SDS-PAGE). Proteins purified in this way were fractionated by SDS-PAGE and transferred to Hybond-P membranes (Amersham, Bucks., UK) that were probed using antibodies against Ser^221^-, Ser^327^- or Thr^246^-phosphorylated Nedd4-2. Blots were then stripped and re-probed using antibodies against the full length protein. This method allowed the recovery of two proteins with molecular weights (~105 kDa and 125 kDa) consistent with Nedd4-2 expression (Boase et al., 2011), and initial experiments confirmed that the recovery of these proteins was dependent upon the inclusion of anti Nedd4-2 in the immunoprecipitation reaction.

### 2.4 Surface expression of ENaC subunits

Control/stimulated cells were exposed (1 hour at 4 °C with gentle agitation) to 10 mM sulfosuccinimidyl-2-(biotinamido)-ethyl-1,3′dithioproprionate (EZ-Link Sulfo-NHS-SS-Biotin, Pierce, Fischer Scientific, UK) to attach a cleavable biotin moiety to surface-exposed proteins. The biotinylation reaction was then terminated (100 mM ice cold glycine), the cells lysed and aliquots (0.5 mg) of extracted protein incubated (1 h at room temperature) with streptavidin-coated agarose beads in order to isolate the biotinylated, surface proteins. These beads were then washed and biotinylated proteins recovered by heating in SDS-PAGE sample buffer. Changes to the surface abundance of α-, β- and γ-ENaC were monitored by subjecting the biotinylated proteins to Western analysis using antibodies against these channel subunits. A full account of this method, which allows surface-exposed proteins to be isolated with ~95% purity, is presented elsewhere (Watt et al., 2012).

### 2.5 Assay of SGK1/PKA activity

Aliquots (40 µg) of protein from control / stimulated cells were fractionated by SDS-PAGE and subject to Western analysis using antibodies against the Thr^346/356/366^-phosphorylated and total NDRG1, and Ser^133^-phosphorylated and total CREB. NDRG1-Thr^346/356/366^ is phosphorylated by SGK1 but not by other, closely related kinases, and increased phosphorylation of these residues must therefore reflect an increase in the activity of this kinase (Inglis et al., 2009; Murray et al., 2004, 2005). Similarly, increased phosphorylation of CREB-Ser^133^ was assumed to reflect increased PKA activity since this residue is an archetypical PKA substrate.

### 2.6 Transepithelial Na^+^ transport

Cells grown to confluence on Snapwell membrane were mounted in Ussing chambers where the current required to hold the transepithelial voltage at 0 mV was monitored as an indicator of active ion transport (Ussing, 1960). In all such experiments, the apical and basolateral sides of the cultured epithelial layers were each bathed with 15 ml of bicarbonate buffered Ringer solution (composition in mM: NaCl, 112; NaHCO_3_, 25; KCl, 4.7; MgSO_4_, 1.2; KH_2_PO_4_, 1.2; CaCl_2_, 2.5 and d-glucose, 11.6, pH 7.3–7.5 when bubbled with 5% CO_2_) which was continually circulated by bubbling with 5% CO_2_/95% O_2_. The current generated by control / stimulated cells was monitored for 30 min before amiloride (10 µM) was added to the apical bath in order to block ENaC. The amiloride-sensitive component of the transepithelial current (*I*_Amil_) was quantified as an indicator of electrogenic Na^+^ absorption.

### 2.7 Data analysis/experimental design

Control / experimental cells were derived from the same culture flasks and subject to an identical series of solution changes. Control cells were always exposed to solvent vehicle and proteins from control/experimental cells were extracted and processed in parallel using identical reagents. None of the effects reported here can thus be attributed to non-specific actions (*e.g*. exposure to solvent vehicle). For quantitative analysis, a computer scanner was used to produce image files of each blot, and a standard software package (ImageJ) then used to quantify the optical density of the bands corresponding to control/experimental protein. Background subtraction was achieved by subtracting the optical density of an identically sized area of clear gel immediately adjacent to the region of interest. To present the pooled data from a series of experiments, individual data values were normalised to the mean density derived by analysis of all control samples within a particular experimental group (i.e. the value derived from the entire series of experiments), and data are therefore shown as fold change over control. Minor adjustments were made to the contrast / brightness of presented images but these changes were applied to the entire image and did not enhance, obscure, move or introduce any feature. Cited molecular weights were estimated by reference to a series of protein standards. The results of experiments that explored the effects of a single test substance were analysed using Student׳s *t*-test, whilst the data from experiments that followed more complex protocols were analysed by one way analysis of variance (ANOVA) / Bonferroni post hoc test. Data are shown as mean±S.E.M, and values of *n* denote the number of independent experiments.

## 3 Results

### 3.1 Effects of dexamethasone/cAMP agonists on SGK1 and PKA

**Fig. 1:**
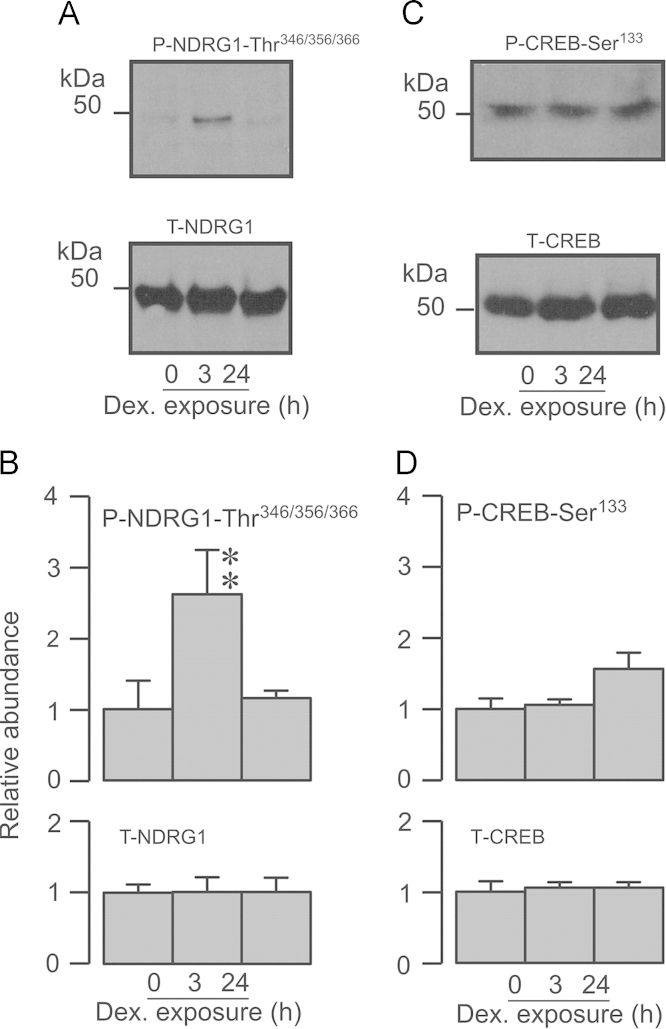
Effects of dexamethasone upon PKA and SGK1. Control cells were maintained in hormone-free medium for 24 h whilst dexamethasone-stimulated cells were exposed to this synthetic glucocorticoid (0.2 µM) for 24 h or 3 h. All cells were then lysed and aliquots (40 µg) of extracted protein subject to Western analysis. (A) Typical blots showing the effects of dexamethasone upon the abundance of Thr^346/356/366^-phosphorylated (upper panel) and total (lower panel) NDRG1. (B) Densitometric analysis showing the pooled data (*n*=5). (C) Typical Western blots showing the effects of dexamethasone upon the abundance of Ser^133^-phosphorylated (upper panel) and total (lower panel) CREB. (D) Densitometric analysis showing the pooled data (*n*=5). Asterixes denote statistically significant difference from the control values measured in hormone-deprived cells (^⁎^^⁎^*P*<0.02, one- way ANOVA/Bonferroni *post-hoc* test). All data are mean±S.E.M.

**Fig. 2:**
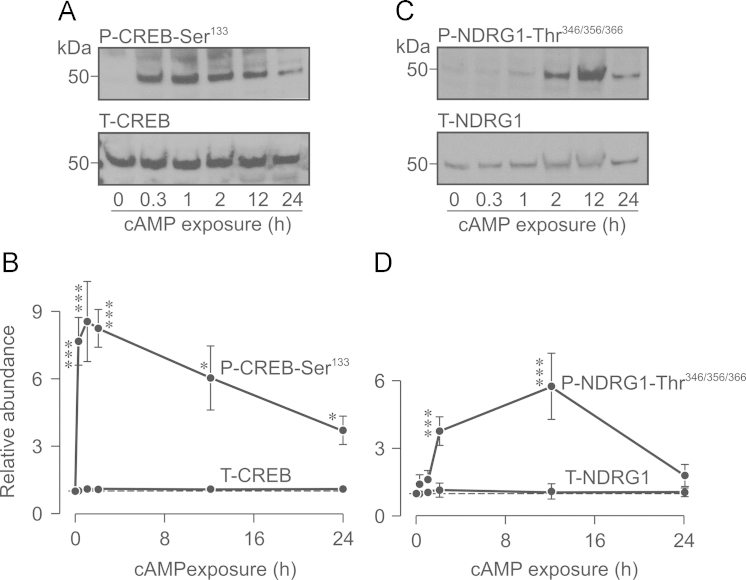
Activation of PKA and SGK1 by cAMP agonists. Glucocorticoid-deprived cells were exposed (0–24 h) to a cocktail of compounds that promote activation of cAMP-dependent signalling pathways and 40 µg aliquots of extracted protein then subject to Western analysis. (A) Typical Western blots showing the effects of cAMP agonists upon the abundance of Ser^133^-phosphorylated (upper panel) and total (lower panel) CREB. (B) Densitometric analysis showing the pooled data from 5 such experiments. (C) Western blots showing the effects of cAMP-agonists upon the abundance of Thr^346/356/366^-phosphorylated (upper panel) and total (lower panel) NDRG1. (D) Densitometric analysis showing the pooled data (*n*=5). Asterixes denote statistically significant deviations from control (^⁎^, *P*<0.05; ^⁎^^⁎^^⁎^*P*<0.01, one-way ANOVA/Bonferroni *post-hoc* test).

Brief (3 h) exposure to dexamethasone (0.2 µM) increased the abundance of Thr^346/356/366^-phosphorylated NDRG1 but did not alter the overall NDRG1 expression level (Fig. 1A, B) indicating (see Methods) activation of SGK1. Parallel studies of cells exposed to this synthetic glucocorticoid for 24 h showed that this response was not sustained and these data thus confirm (see also Inglis et al., 2009; Watt et al., 2012) that glucocorticoids evoke transient activation of SGK1 in H441 cells. Dexamethasone (0.2 µM, 3 h or 24 h) had no effect upon the abundance of Ser^133^-phosphorylated or total CREB (Fig. 1C, D) and this synthetic hormone thus has no effect upon PKA. Exposing glucocorticoid-deprived cells to cAMP agonists (see Methods), on the other hand, increased the abundance of Ser^133^-phosphorylated CREB without altering the overall CREB expression level (Fig. 2A, B), and these substances therefore activate PKA. This response peaked after ~20 min and, whilst there was some subsequent decline, increased activity persisted for at least 24 h (Fig. 2A, B). Exposure to cAMP agonists also increased the abundance of the Thr^346/356/366^-phosphorylated NDRG1 with no effect upon overall NDRG1 expression (Fig. 2C, D) indicating activation of SGK1. This response did not become apparent until ~2 h and peaked after ~12 h (Fig. 2C, D), and the cAMP-induced activation of SGK1 therefore occurs more slowly than the activation of PKA. Brief (3 h) exposure to dexamethasone thus provides a way of activating SGK1 independently of PKA, whilst 20 min exposure to cAMP agonists causes selective activation of PKA. Subsequent experiments therefore explored the effects of these manoeuvres upon the phosphorylation of Nedd4-2 and the surface abundance of ENaC subunits.

### 3.2 Effects of dexamethasone on Nedd4-2

**Fig. 3:**
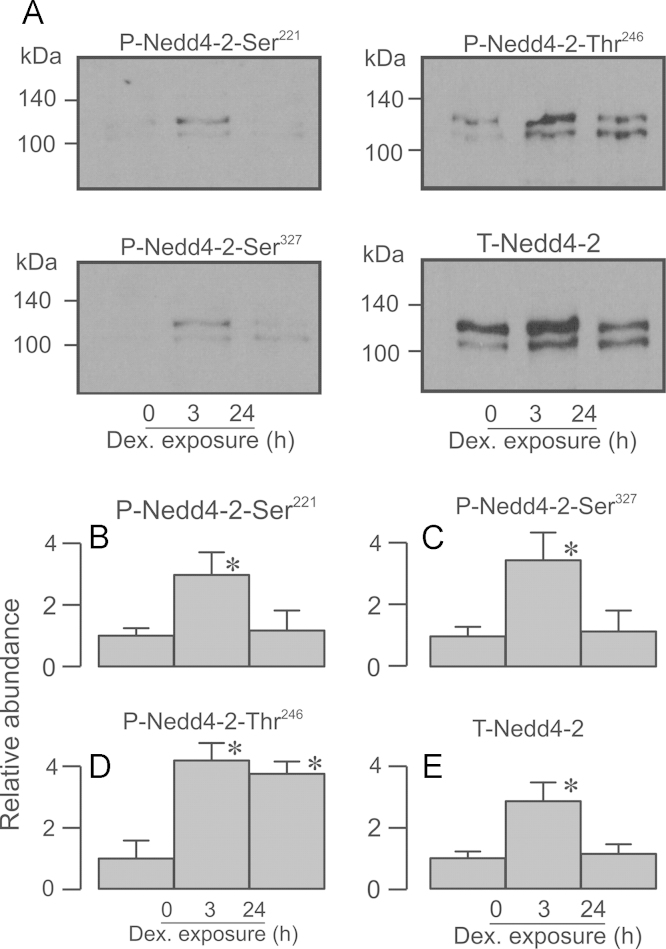
Effects of dexamethasone upon the phosphorylation/abundance of Nedd4-2. Nedd4-2 was immunopurified from glucocorticoid-deprived cells and from cells exposed to 0.2 µM dexamethasone for 3 h or 24 h. (A) Typical Western blots showing the effects of dexamethasone stimulation (3 h and 24 h) upon the abundance of the Ser^221^-phosphorylated, Ser^327^-phosphorylated, Thr^246^-phosphorylated and total Nedd4-2. (B–E) Densitometric analysis showing the pooled data from the entire series of experiments (*n*=8). Asterixes denote statistically significant differences from the values measured in hormone-deprived cells (^⁎^*P*<0.05, one-way ANOVA/Bonferroni *post-hoc* test). All data are mean±S.E.M.

**Fig. 4:**
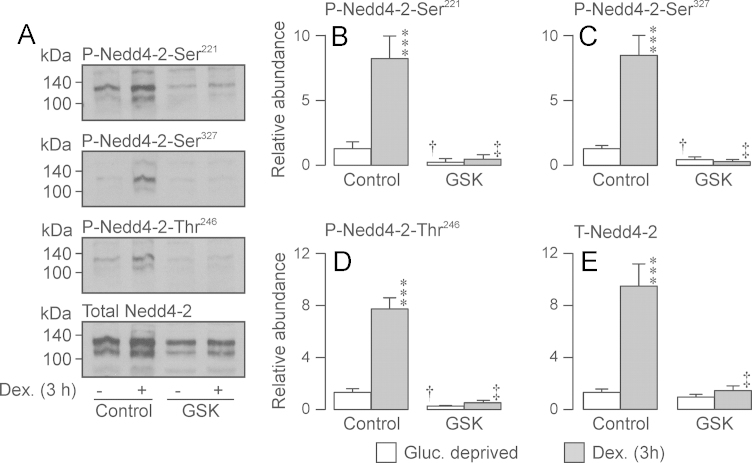
Effects of GSK650394 upon the dexamethasone-induced changes to the phosphorylation / abundance of Nedd4-2. Nedd4-2 was immunopurified from control and GSK650394-treatred (10 µM, 3 h) cells that were either maintained in hormone-free medium or exposed to 0.2 *µ*M dexamethasone for 3 h. (A) Typical Western blots showing the effects of dexamethasone and/or GSK650394 upon the cellular abundance of Ser^221^-phosphorylated, Ser^327^-phosphorylated, Thr^246^-phosphorylated and total Nedd4-2. (B–E) Densitometric analysis showing the pooled results of the entire series of experiments (*n*=4). Asterixes denote statistically significant effects of dexamethasone (^⁎^*P*<0.05, ^⁎^^⁎^^⁎^*P*<0.01) whilst daggers († P<0.05, ‡ *P*<0.01) indicate significant effects of GSK650394 (one-way ANOVA / Bonferroni test). All data are mean±S.E.M.

Brief (3 h) exposure to 0.2 µM dexamethasone increased the abundance of the Ser^221^-, Ser^327^- and Thr^246^-phosphorylated Nedd4-2 but also increased the overall Nedd4-2 expression level (Fig. 3). Since overall abundance of Nedd4-2 was assessed by stripping / re-probing blots that had first been probed with a phospho-peptide specific antibody (see Methods), we were concerned that this apparent increase may be an artefact caused by the incomplete removal of these antibodies from the blots. However, additional experiments in which blots were simply probed with anti-Nedd4-2 provided virtually identical data and this possibility can thus be excluded. To quantify the effects of dexamethasone upon the phosphorylation status of Nedd4-2-Ser^221^, -Ser^327^ and -Thr^246^, the data from all experiments in which cells were exposed to dexamethasone for 3 h (*i.e*. those shown in Fig. 3, Fig. 4 and Fig. 6) were further analysed by normalising the increased abundance of each phosphoprotein to the corresponding increase in total expression. This analysis showed that the effects of dexamethasone upon the abundance of Ser^221^-, Ser^327^- and Thr^246^-phosphorylated Nedd4-2 were matched by the increase in overall expression (Ser^221^: 0.772±0.204, Ser^327^: 0.909±0.194, Thr^246^: 1.104±0.193). Activating SGK by brief exposure to dexamethasone thus increases Nedd4-2 abundance but has no effect upon the relative phosphorylation of these residues. The SGK inhibitor, GSK650394 (3 h, 10 µM) reduced the abundance of Ser^221^-, Ser^327^- and Thr^248^-phosphorylated Nedd4-2 but did not alter the overall expression level. Inhibition of SGK thus suppresses the basal phosphorylation of these residues. GSK650394 also abolished the effects of dexamethasone (3 h, 0.2 *µ*M) upon the abundance of phosphorylated and total forms of this protein (Fig. 4). Prolonged (24 h) exposure to 0.2 µM dexamethasone had no effect upon the abundance of Ser^221^-phosphorylated, Ser^327^-phosphorylated or total Nedd4-2 (Fig. 3) and the effects on these residues are therefore transient. However, increased expression of Thr^246^-phosphorylated Nedd4-2 persisted after 24 h. Since the overall abundance of Nedd4-2 had returned to its basal value by this time (Fig. 3), chronic exposure to dexamethasone must cause sustained phosphorylation of Nedd4-2-Thr^246^. In addition, these data indicate that phosphorylation of Ser^221^/Ser^327^ must be important in the regulation of Nedd4-2 abundance.

### 3.3 Effects of dexamethasone on the surface abundance of ENaC subunits

**Fig. 5:**
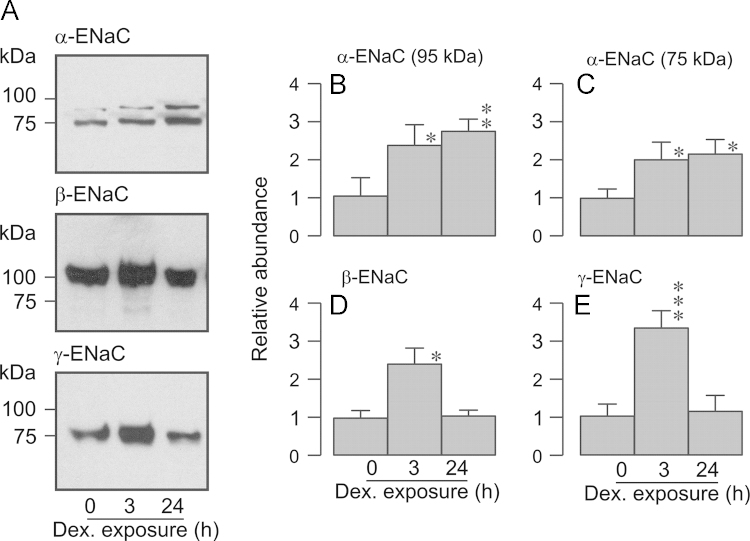
Effects of dexamethasone upon the surface abundance of α-, β- and γ-ENaC. Surface-exposed proteins from glucocorticoid-deprived cells and from cells exposed to 0.2 µM dexamethasone for 3 h or 24 h were subject to Western analysis using antibodies against α-, β- or γ-ENaC. (A) Typical Western blots showing the effects of dexamethasone (3 h and 24 h) upon the surface abundance of these channel subunits. (B) Densitometric analysis showing the results of the entire series of experiments (*n*=7). Asterixes denote statistically significant differences from the values measured in hormone-deprived cells (^⁎^*P*<0.05, ^⁎^^⁎^^⁎^*P*<0.001one-way ANOVA / Bonferroni *post-hoc* test). All data are mean±S.E.M.

Analysis of surface-exposed proteins confirmed (Watt et al., 2012) that α-, β- and γ-ENaC were present in the membranes of glucocorticoid-deprived cells (Fig. 5). The antibody against *α*-ENaC consistently detected two bands (~75 kDa and~95 kDa) whilst β- and γ-ENaC were present as single bands (~100 KDa and 70 kDa respectively). The fact that the antibody against *α*-ENaC consistently (see also Watt et al., 2012) identifies two bands is consistent with the hypothesis that the post-translational processing of *α*-ENaC involves cleavage by intracellular and extracellular proteases; indeed, such proteolytic cleavage is a central part of the mechanism that allows ENaC to become active (Rossier and Stutts, 2009; Vallet et al., 1997). Since these proteases act at different sites, the processing of this channel subunit is known to generate a complex pattern of polypeptide fragments. The present analyses were undertaken using antibodies directed against N-terminal residues, and the apparent pattern of cleavage that we report here is therefore consistent with that described in earlier studies (Rossier and Stutts, 2009). However, although it is clear that γ-ENaC is also subject to such proteolytic cleavage (Haerteis et al., 2012; Rossier and Stutts, 2009), we only identified a single, 70 kDa band in the present study. Whilst earlier studies of H441 cells, which were undertaken using the same antibodies, did reveal a second band whose apparent molecular weight (~90 kDa), was consistent with the uncleaved form of γ-ENaC, the heavier form of this protein was only detected by analysing proteins that had simply been extracted from intact cells. Analyses of surface exposed proteins (present study, Watt et al., 2012), therefore indicate that this ‘full length’ form of γ-ENaC is not present in the plasma membrane.

Acute (3 h) exposure to dexamethasone increased the surface abundance all three subunits (Fig. 5) and, whilst the effects on α-ENaC persisted after 24 h, the surface abundance of β- and γ-ENaC returned to their basal levels by this time (Fig. 5).

### 3.4 Effects of cAMP agonists upon Nedd4-2 and ENaC in glucocorticoid-deprived cells

**Fig. 6:**
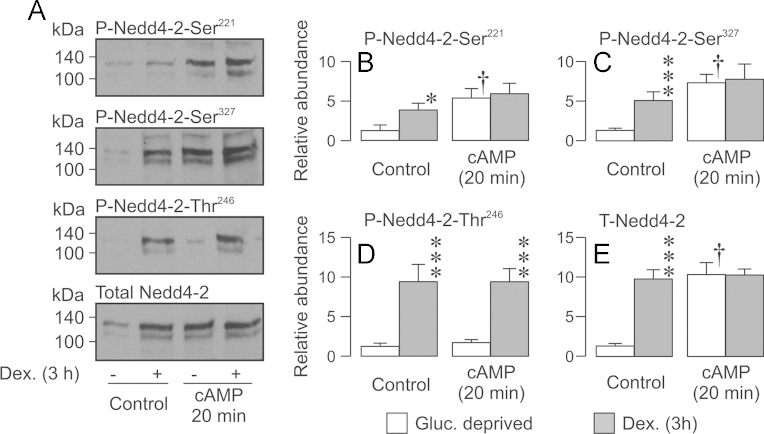
Effects of 0.2 µM dexamethasone (3 h) and / or cAMP agonists (20 min) and upon the phosphorylation / abundance of Nedd4-2. Nedd4-2 was immunopurified from glucocorticoid-deprived and dexamethasone-stimulated (0.2 µM, 3 h) cells that had been either maintained under control conditions or exposed to cAMP agonists for the final 20 min of this incubation period. (A) Typical Western blots showing the effects of cAMP and dexamethasone upon the cellular abundance of Ser^221^-phosphorylated, Ser^327^-phosphorylated, Thr^246^-phosphorylated and total Nedd4-2. (B–E) Densitometric analysis showing the pooled data (*n*=3) from the entire series of experiments. Asterixes denote statistically significant effects of dexamethasone (^⁎^*P*<0.05, ^⁎^^⁎^^⁎^ P<0.01) whilst daggers show significant († *P*<0.05) effects of cAMP agonists (one-way ANOVA / Bonferroni test). All data are mean±S.E.M.

**Fig. 7:**
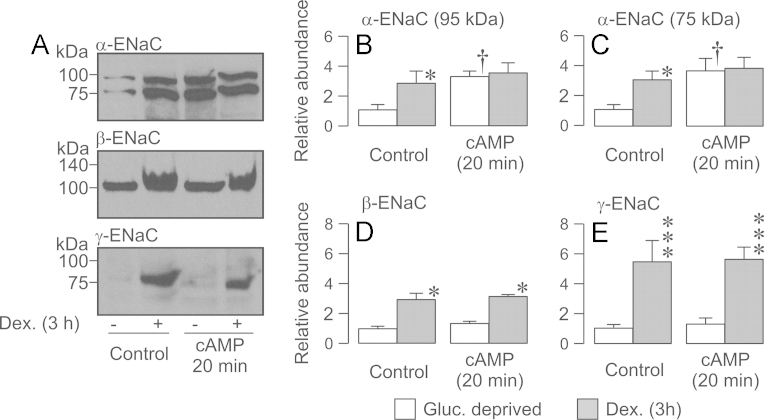
Effects of 0.2 µM dexamethasone (3 h) and/or cAMP agonists (20 min) upon the surface abundance of α-, β- and γ-ENaC. Surface-exposed proteins were isolated from glucocorticoid-deprived and dexamethasone-stimulated cells that were either maintained under control conditions or exposed to the cAMP agonists for the final 20 min of this incubation period. (A) Typical Western blots showing the effects of dexamethasone and/or cAMP agonists upon the surface abundance of α-, β- and γ-ENaC. (B–E) Densitometric analysis showing the pooled data (mean±S.E.M.) from the entire series of experiments. Asterixes denote statistically significant effects of dexamethasone (^⁎^*P*<0.05, ^⁎^^⁎^^⁎^ P<0.01) whilst daggers show significant († *P*<0.05) effects of cAMP agonists (one-way ANOVA/Bonferroni *post-hoc* test). All data are mean±S.E.M.

The control data in Fig. 6 confirm that brief (3 h) exposure to 0.2 µM dexamethasone increases the abundance of Ser^221^-phosphorylated, Ser^327^-phosphorylated, Thr^246^-phosphorylated and total Nedd4-2. Parallel studies of cells acutely (20 min) exposed to cAMP agonists revealed increased abundance of Ser^221^-phosphorylated and Ser^327^-phosphorylated Nedd4-2. Whilst these effects were accompanied by a clear increase in overall Nedd4-2 abundance, the cAMP agonists had no effect upon the abundance of Thr^246^-phosphorylated Nedd4-2 (Fig. 6). Experiments in which dexamethasone-stimulated (0.2 µM, 3 h) cells were exposed to cAMP agonists for the final 20 min of this incubation period showed that activating PKA has no additional effect upon the phosphorylation / abundance of Nedd4-2 (Fig. 6). We used a similar experimental protocol to explore the effects of SGK1 and / or PKA upon the surface abundance of α-, β- and γ-ENaC. Brief (3 h) exposure to 0.2 µM dexamethasone increased the surface abundance of each subunit (see also Watt et al., 2012) whilst brief (20 min) exposure to cAMP agonists increased the surface abundance of *α*-ENaC but had no effect upon the amounts of β- and γ-ENaC in the membrane (Fig. 7). Analysis of protein from dexamethasone-stimulated (0.2 µM, 3 h) cells that were also exposed to cAMP agonists for 20 min showed that PKA activation had no additional effect upon the surface abundance of α-, β- or γ-ENaC (Fig. 7). Acutely activating SGK1 and PKA in glucocorticoid-deprived cells therefore have different effects upon the phosphorylation of Nedd4-2 and the surface expression of ENaC subunits.

### 3.5 PKA/SGK1 activity in cells chronically (24 h) exposed to dexamethasone

**Fig. 8:**
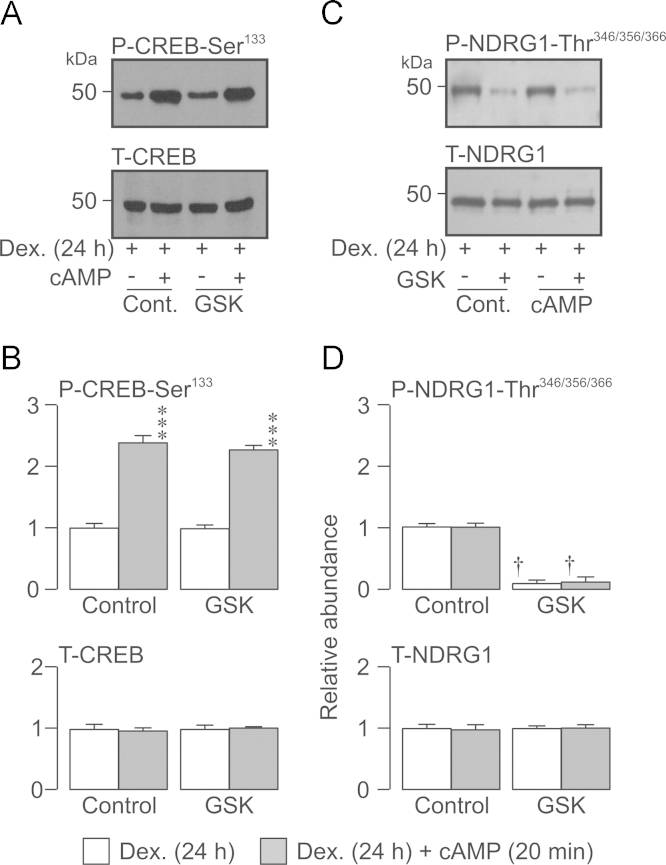
Effects of GSK650394 and/or cAMP agonists upon PKA and SGK1 in cells chronically (24 h) exposed to 0.2 µM dexamethasone. Dexamethasone-treated (0.2 µM, 24 h) cells (*n*=3) were exposed to cAMP agonists 20 min under standard conditions and in the presence of GSK650394 (10 *µ*M, 3 h) and aliquots (40 µg) of extracted protein then subject to Western analysis. (A) Typical Western blots showing the effects of GSK650394 and/or cAMP agonists upon the abundance of Ser^133^-phosphorylated and total CREB. (B) Densitometric analysis showing pooled data (*n*=3) from the entire series of experiments. (C) Typical Western blots showing the effects of GSK650394 and/or cAMP agonists upon the abundance of Thr^346/356/366^-phosphorylated (upper panels) and total NDRG1 (lower panels). (D) Densitometric analysis showing pooled data (*n*=3) from the entire series of experiments. Asterixes denote statistically significant effects of cAMP agonists (^⁎^^⁎^^⁎^*P*<0.01) whilst daggers (†, *P*<0.05) denote statistically significant effects of GSK650394 (one-way ANOVA / Bonferroni *post-hoc* test). All data are mean±S.E.M.

Applying cAMP agonists (20 min) to cells that had been exposed to 0.2 *µ*M dexamethasone for 24 h increased the abundance of Ser^133^-phosphorylated CREB with no effect upon overall CREB expression (Fig. 8A, B). Since these treatments had no effect upon the phosphorylation status of NDRG1-Thr^346/356/366^ (Fig. 8C, D), these data show that the cAMP agonists selectively activate PKA. Applying GSK650394 (10 µM, 3 h) to dexamethasone-treated (24 h) cells suppressed the phosphorylation of NDRG1-Thr^346/356/366^, but had no effect upon the cAMP-induced phosphorylation of CREB-Ser^133^. As both dexamethasone (24 h) and cAMP agonists (20 min) did not elevate NDRG1-Thr^346/356/366^ phosphorylation (Fig. 1) these findings establish that GSK650394 inhibits basal SGK1 activity but does not affect the control of PKA.

### 3.6 Effects of cAMP agonists upon Nedd4-2 and ENaC in dexamethasone-treated (24 h) cells

**Fig. 9:**
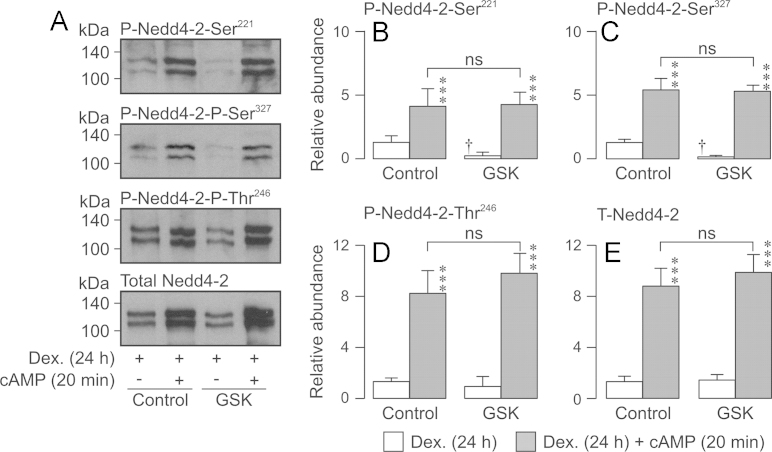
Effect of cAMP agonists and / or GSK650394 upon the phosphorylation /abundance of Nedd4-2 in dexamethasone-treated (0.2 µM, 24 h) cells. Proteins immunopurified from dexamethasone-treated (0.2 µM, 24 h) cells either maintained under standard conditions or exposed to cAMP agonists for 20 min (*n*=3). Experiments were undertaken using both control and GSK650394-treated (10 µM, 3 h) cells. (A) Typical Western blots showing the effects of cAMP and / or GSK650394 upon the cellular abundance of Ser^221^-phosphorylated, Ser^327^-phosphorylated, Thr^246^-phosphorylated and total Nedd4-2. (B–E) Densitometric analysis showing the pooled data (*n*=3) from the entire series of experiments. Asterixes denote statistically significant effects of cAMP agonists (^⁎^^⁎^^⁎^*P*<0.01) whilst daggers (†, *P*<0.05) denote statistically significant effects of GSK650394 (one-way ANOVA/Bonferroni *post-hoc* test). All data are mean±S.E.M.

Applying cAMP agonists (20 min) to dexamethasone-treated (24 h) cells increased the abundance of Ser^221^- and Ser^327^-phosphorylated Nedd4-2, increased the overall Nedd4-2 expression level and also increased the abundance of Thr^246^-phosphorylated Nedd4-2 (Fig. 9A, D). Since the effect on Nedd4-2-Thr^246^ was not seen in glucocorticoid-deprived cells, chronic (24 h) exposure to dexamethasone must allow PKA to phosphorylate this residue. Applying GSK650394 (10 µM, 3 h) to dexamethasone-treated (24 h) cells reduced the abundance of the Ser^221^- and Ser^327^-phosphorylated Nedd4-2 without altering the overall Nedd4-2 expression level indicating that inhibition of SGK1 suppresses the basal phosphorylation of these residues. Although there was a reduction in the abundance of Thr^246^-phosphorylated Nedd4-2, this effect was not statistically significant (Fig. 9D). In the presence of GSK650394, cAMP agonists (20 min) increased the abundance of Ser^221^-phosphorylated, Ser^327^-phosphorylated, Thr^246^-phosphorylated and total Nedd4-2 to the levels seen in control cells (Fig. 9). The effects of PKA upon the expression / phosphorylation of Nedd4-2 in dexamethasone-treated (24 h) cells therefore occur independently of SGK1.

**Fig. 10:**
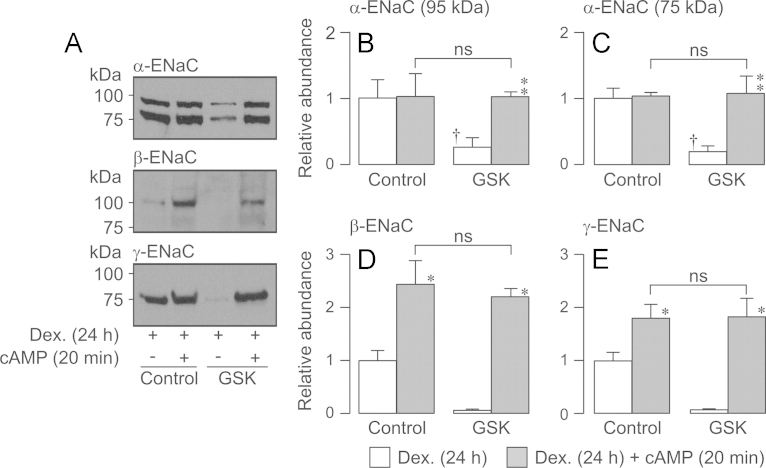
Effect of cAMP agonists and / or GSK650394 upon the surface abundance of α-, β- and γ-ENaC in dexamethasone-treated (0.2 µM, 24 h) cells. Surface-exposed proteins were isolated from dexamethasone-treated (0.2 µM, 24 h) cells that had been maintained under standard conditions or exposed to cAMP agonists for 20 min. Experiments were undertaken both under control conditions and in the presence of GSK650394 (10 µM, 3 h). (A) Typical Western blots showing the effects of cAMP and/or GSK650394 upon the surface abundance of α-, *β*- and *γ*-ENaC. (B–E) Densitometric analysis showing the pooled data (*n*=3) from the entire series of experiments. Asterixes denote statistically significant effects of cAMP agonists (^⁎^^⁎^^⁎^*P*<0.01) whilst daggers (†, *P*<0.05) denote statistically significant effects of GSK650394 (one-way ANOVA / Bonferroni *post-hoc* test). All data are mean±S.E.M.

**Fig. 11:**
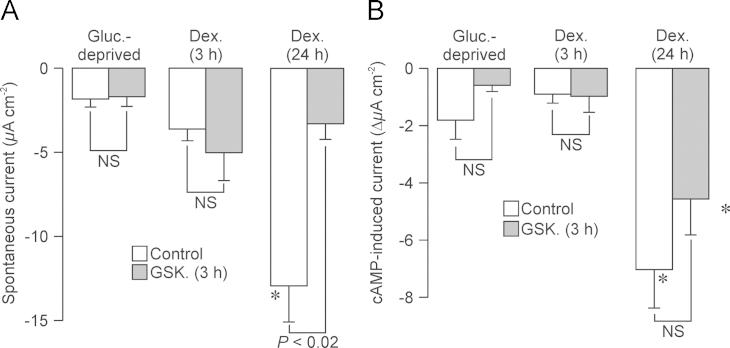
Effects of cAMP agonists / GSK650394 on electrogenic Na^+^ absorption in glucocorticoid-deprived and dexamethasone-treated cells. (A) Cells grown to confluence on permeable supports were either deprived of glucocorticoids for 24 h, or exposed to 0.2 µM dexamethasone for 3 h or 24 h. Control/GSK650394-treated (10 µM, 3 h) cells were then mounted in Ussing chambers and amiloride-sensitive short circuit current (*I*_Amil_) quantified as an indicator of the electrogenic Na^+^ transport rate. (B) Cyclic AMP agonist-induced increases in *I*_Amil_ above the levels quantified in parallel studies of unstimulated cells that were age matched, at identical passage number and had been maintained in Ussing chambers for identical time periods. Presented values of *P* denote statistically significant effects of GSK650394 whilst asterixes denote significant effects of dexamethasone (*P*<0.05, one-way ANOVA/Bonferroni *post-hoc* test). All data are mean±S.E.M.

Subsequent experiments used this stimulation protocol to explore the effects of PKA upon the surface expression of α-, β- and γ-ENaC in dexamethasone-treated (24 h) cells. All three ENaC subunits were present in the membranes of control cells and, whilst the cAMP agonists (20 min) had no effect upon the surface expression of α-ENaC (Fig. 10A, B, C), they did increase the amounts of β- (Fig. 10A, D) and γ-ENaC (Fig. 10A, E) in the membrane. Since these drugs did not alter the surface abundance of β- and γ-ENaC in glucocorticoid-deprived cells (Fig. 7), the present data show that chronic (24 h) exposure to dexamethasone allows PKA to control the surface expression of these subunits. Exposing dexamethasone-treated cells to GSK650394 reduced the surface abundance of α- (Fig. 11A, B, C), β- (Fig. 11 A, D) and γ-ENaC (Fig. 10A, E). However, despite this clear and consistent finding, activating PKA under these conditions increased the abundance of each subunit to the level seen in control cells (Fig. 11). The effects of cAMP agonists upon the surface expression of ENaC subunits are therefore independent of SGK1.

### 3.7 Electrogenic Na^+^ transport

Electrometric studies of cells grown to confluence on permeable supports (see Methods) showed that glucocorticoid-deprived cells generated ~2 *µ*A cm^−2^ of amiloride-sensitive short circuit current (*I*_Amil_). Moreover, although the current recorded from cells that had been exposed to 0.2 µM dexamethasone for 3 h was slightly larger, this difference was not statistically significant and we therefore conclude that such brief exposure (3 h) to dexamethasone has no discernible effect upon the rate of electrogenic Na^+^ transport (Fig. 11 A). Prolonged (24 h) exposure to 0.2 µM dexamethasone, on the other hand, caused a clear increase (~8 fold) in the magnitude of *I*_Amil_ (Fig. 11 A) indicating a clear stimulation of Na^+^ transport. Moreover, whilst GSK650394 (10 *µ*M, 3 h) had no discernible effect upon *I*_Amil_ in glucocorticoid-deprived cells and in cells that had been briefly (3 h) exposed to 0.2 *µ*M dexamethasone (Fig. 11A), this inhibitor of SGK1 consistently reduced the current recorded from dexamethasone-treated cells to the level seen glucocortiocoid-deprived cells (Fig. 11A). Chronic exposure to dexamethasone therefore induces increased Na^+^ transport via a mechanism dependent upon SGK1.

Whilst acute exposure to cAMP agonists enhanced the current generated by glucocorticoid-deprived cells, this response was very small (~2 µA cm^−2^) and did not reach statistical significance; it is therefore clear that cAMP agonists have no measurable effect upon the small currents recorded under these conditions. Essentially identical data were recorded from cells that had been exposed to dexamethasone for only 3 h, and such brief exposure to this synthetic glucocorticoid therefore has no effect upon this response to cAMP agonists. However, when applied to cells that had been exposed to dexamethasone for ~24 h, the cAMP agonists consistently increased the magnitude of *I*_Amil_ (Δ*I*_Amil_~5 µA cm^−2^, Fig. 11B) indicating a clear stimulation of Na^+^ transport. It is now clear that chronic exposure to dexamethasone enhances the effects of cAMP upon electrogenic Na^+^ transport. GSK650394 (10 µM, 3 h) had no effect upon the small responses to cAMP agonists seen in glucocorticoid-deprived cells and in cells exposed to dexamethasone for 3 h, and also failed to modify the much larger responses seen in cells exposed to dexamethasone for 24 h (Fig. 11B).

## 4 Discussion

### 4.1 Experimentally-induced activation of SGK1 and PKA

Dexamethasone caused transient activation of SGK1 (see also Inglis et al., 2009; Watt et al., 2012) but not PKA whilst cAMP agonists activated both kinases. The additional effect of cAMP accords with earlier data which suggest that activating PKA increases cellular SGK1 activity by evoking SGK1 gene expression and by phosphorylating SGK1-Thr^389^. It has therefore been suggested that the PKA/cAMP-coupled agonists might stimulate Na^+^ transport via a mechanism dependent upon SGK1 (Gonzalez-Robayna et al., 2000; Perrotti et al., 2001; Thomas et al., 2004; Vasquez et al., 2008). However, not all data support this view (see for example Inglis et al., 2009; Mansley and Wilson, 2010; Snyder et al., 2004a) and we now show the cAMP-induced activation of PKA is rapid whilst the increase in SGK1 activity involves a latency of ~2 h. In contrast, functional studies clearly show that PKA-coupled agonists stimulate Na^+^ transport within 5–10 min (Clunes et al., 2004; Lazrak and Matalon, 2003; Mansley and Wilson, 2010; Morris and Schafer, 2002; Ramminger et al., 2004; Thomas et al., 2004). Whilst PKA-induced activation of SGK1 (Gonzalez-Robayna et al., 2000; Perrotti et al., 2001; Thomas et al., 2004; Vasquez et al., 2008) may contribute to the responses to maintained stimulation, it cannot explain the rapid responses seen in human airway epithelia (Clunes et al., 2004; Lazrak and Matalon, 2003; Ramminger et al., 2004; Thomas et al., 2004).

### 4.2 Changes to the overall abundance of Nedd4-2

Selectively activating SGK1 by brief (3 h) exposure to dexamethasone increased the abundance of Ser^221^-, Ser^327^- and Thr^246^-phosphorylated Nedd4-2 indicating that these residues are SGK1 substrates (Debonneville et al., 2001; Snyder et al., 2004a; Snyder et al., 2002). However, these effects were accompanied by clear increases in overall Nedd4-2 abundance and, since the magnitudes of the two responses were similar, activating SGK1 did not change the relative phosphorylation status of Nedd4-2-Ser^221^, -Ser^327^ or -Thr^246^. It is important to stress that this does not imply that phosphorylation of these residues does not take place. Indeed, phosphorylation must occur if the phosphorylation status of each residue is to be maintained despite an increase in overall abundance. In this context it is relevant that studies of *Xenopus* Nedd4-2 show that phosphorylation of a residue equivalent to human Nedd4-2-Ser^327^ blocks the degradation of this protein (Chandran et al., 2011). Moreover, since the degradation of Nedd4-2 is normally rapid (Bruce et al., 2008), it has been suggested that the phosphorylation of Nedd4-2 at Ser^327^ might increase the stability of the protein and thus increase its abundance (Chandran et al., 2011). Whilst the present data are consistent with this hypothesis, we cannot exclude the possible that other mechanisms may underlie the observed changes to the overall abundance of Nedd4-2. For example, the present data would also be consistent with a model in which dexamethasone promoted the *de novo* synthesis of Nedd4-2 protein which was then phosphorylated by SGK1. More detailed studies using fully quantitative methods are therefore needed to establish the physiological basis of this effect.

### 4.3 Acute activation of PKA or SGK1 has different effects upon Nedd4-2 and ENaC

Whilst acute (3 h) activation of SGK1 caused phosphorylation of Nedd4-2-Ser^221^, -Ser^327^ and -Thr^246^, activating PKA in glucocorticoid-deprived cells caused phosphorylation of Nedd4-Ser^221^ and Nedd4-Ser^327^ but not Nedd4-2-Thr^246^. Whilst these findings suggest that Nedd-4-2-Thr^246^ is not be phosphorylated by PKA, we cannot formally establish this mechanism since similar data would be obtained if the cAMP agonists were able to promote the dephosphoryation of this residue. However, it is relevant our findings do accord with data derived from heterologously-expressed Nedd4-2 which do show that Thr^246^ is a substrate for SGK1 but not PKA (Debonneville et al., 2001; Snyder et al., 2002, 2004a). Moreover, whilst acute activation of SGK1 increased the surface abundance of α-, β- and γ-ENaC, activating PKA increased the abundance of *α*-ENaC with no effect upon β- and γ-ENaC. Activating PKA in glucocorticoid-deprived cells therefore fails to induce a coordinated increase in the surface expression of α-, β- and γ-ENaC, and this is consistent with the idea that control over the surface abundance of ENaC subunits requires the phosphorylation of Nedd4-2 at both Ser^327^ and Thr^246^ (Chandran et al., 2011; Snyder et al., 2004a).

### 4.4 Acute and chronic effects of dexamethasone

As anticipated (Althaus et al., 2010; Ramminger et al., 2004), prolonged (24 h) exposure to dexamethasone consistently induced a Na^+^ absorbing phenotype. However, whilst the effects of dexamethasone upon SGK1, Nedd4-2 and the surface abundance of ENaC all peaked after ~3 h (present study, Inglis et al., 2009; Watt et al., 2012), such brief exposure to dexamethasone had no discernible effect upon electrogenic Na^+^ transport. Moreover, whilst chronic exposure to dexamethasone consistently evoked Na^+^ transport via an SGK1-dependent mechanism, this stimulus did not cause persistent activation of SGK1 (present study, Inglis et al., 2009; Watt et al., 2012). Furthermore, whilst chronic exposure to dexamethasone caused persistent phosphorylation of Nedd4-2-Thr^246^, the abundance of Ser^221^-phosphorylated, Ser^327^-phosphorylated and total Nedd4-2 had all fallen to the levels seen in glucocorticoid-deprived cells after 24 h exposure to this synthetic hormone. In addition, although prolonged stimulation increased the surface abundance of α-ENaC it had no effect upon β-ENaC or γ-ENaC (see also Watt et al., 2012). This selective increase in the surface abundance of α-ENaC cannot explain the increased Na^+^ transport since acute exposure to cAMP agonists had an essentially identical effect upon the surface abundance of ENaC subunits and yet caused only a very small increase in the rate of Na^+^ transport (see above). The sustained absorption of Na^+^ that is characteristically seen in dexamethasone-treated H441 cells (Althaus et al., 2010; Inglis et al., 2009; Ramminger et al., 2004) therefore occurs independently of maintained phosphorylation of Nedd4-2-Ser^221^ and Nedd4-2Ser^327^, and does not involve a coordinated increase in the surface abundance of α-, β- and γ-ENaC. Nevertheless, GSK650394-mediated inhibition of SGK1 suppressed the phosphorylation of Nedd4-2-Ser^221^ and -Ser^327^ and also reduced the surface abundance of α-ENaC, and these findings show that SGK1 activity is needed to maintain α-ENaC in the membranes. This, in turn, suggests that α-ENaC might contribute to the increased Na^+^ transport.

Whilst our data show that signalling via SGK1 – Nedd4-2 allows control over the surface abundance of ENaC subunits (Boase et al., 2011; Debonneville et al., 2001; Flores et al., 2003, 2005; Kimura et al., 2011; Morris and Schafer, 2002; Snyder et al., 2002, 2004a, 2004b), they also indicate that signalling via this pathway cannot explain how prolonged exposure to glucocorticoids is able to induce a Na^+^ absorbing phenotype. However, earlier studies of heterologously expressed Nedd4-2 suggest that Thr^246^ is of central importance to the control over the surface abundance of ENaC subunits (Chandran et al., 2011; Snyder et al., 2004a) and it is therefore interesting that prolonged exposure to dexamethasone did cause sustained phosphorylation of this residue. Moreover, that fact that this effect persisted in the presence of GSK650394, indicates that this response is maintained independently of SGK1.

### 4.5 Chronic exposure to dexamethasone enhances the effects of activating PKA

Acutely activating PKA in dexamethasone-treated cells promoted phosphorylation of all three residues within Nedd4-2, increased the surface abundance of α-, β- and γ-ENaC, and caused an unambiguous stimulation of Na^+^ transport. Since these responses differ from those seen in glucocorticoid-deprived cells, our data show that, as *well as* inducing a Na^+^ absorbing phenotype, glucocorticoids allow cAMP agonists to control Na^+^ transport via the PKA – Nedd4-2 – ENaC pathway. This situation resembles that seen in the lungs of foetal lambs (Barker et al., 1990; Olver et al., 1986; Walters et al., 1990) and in primary cultures of foetal rat distal lung epithelial cells (Ramminger et al., 2002) where the cAMP-dependent control of Na^+^ transport requires prior exposure to glucocorticoid / thyroid hormones. Moreover, whilst the direct effects of dexamethasone upon Na^+^ transport were abolished by GSK650394, cAMP agonists could still activate the PKA – Nedd4-2 – ENaC pathway in GSK650394-treated cells. Whilst these data accord with earlier observations which suggest that cAMP agonists control Na^+^-transport via a mechanism that is independent of SGK1 (Inglis et al., 2009; Mansley and Wilson, 2010; Snyder et al., 2004a), they also show that SGK1 cannot mediate the permissive effects of dexamethasone which we now report.

### 4.6 Conclusions and significance of present findings

The present studies of endogenously expressed proteins show that dexamethasone activates SGK1, induces phosphorylation of Nedd4-2 and increases the surface abundance of ENaC subunits. However, these effects did not coincide with the stimulation of Na^+^ transport and it is therefore clear that signalling via SGK1 – Nedd4-2 – ENaC cannot account for the enhanced Na^+^ transport seen in glucocorticoid-stimulated cells. Other factors, such as effects on the Na^+^ pump, K^+^ channels and other components of the ENaC channel complex (Barnes, 2011), must therefore be important. Dexamethasone-induced Na^+^ transport was, however, dependent upon SGK1 indicating that this kinase must play a central role. It is therefore interesting that SGK1 appears to activate ENaC by directly phosphorylating residues within *α*-ENaC itself (Diakov and Korbmacher, 2004). However, the most important finding to emerge from the present study was that prolonged exposure to dexamethasone allowed cAMP agonists to evoke phosphorylation of Nedd4-2 at Thr^246^. Earlier studies had indicated that Nedd4-2-Thr^246^ was of critical important to the control of ENaC abundance (Chandran et al., 2011; Snyder et al., 2004a), and it is therefore interesting that dexamethasone also allowed cAMP agonists to induce coordinated increases in the surface abundance of α-, β- and γ-ENaC and to stimulate Na^+^ absorption. As well as inducing a stable, Na^+^ absorbing phenotype, glucocorticoids therefore facilitate signalling via the PKA – Nedd4-2 – ENaC pathway and thus allow cAMP agonists to exert acute control over the rate of Na^+^ absorption.

## Funding

This work was supported by a grant from the MRC (SMW), BBRC (DLB) and by a post graduate student ship from the Government of Malaysia (NASI).
